# A Comparison of the Prognostic Effects of Fine Needle Aspiration and Core Needle Biopsy in Patients with Breast Cancer: A Nationwide Multicenter Prospective Registry

**DOI:** 10.3390/cancers15184638

**Published:** 2023-09-19

**Authors:** Hongki Gwak, Sang Seok Woo, Se Jeong Oh, Jee Ye Kim, Hee-Chul Shin, Hyun Jo Youn, Jung Whan Chun, Dasom Lee, Seong Hwan Kim

**Affiliations:** 1 Division of Thyroid and Breast Surgical Oncology, Department of Surgery, Hwahong Hospital, Suwon 16630, Republic of Korea; hkgwak@gmail.com; 2 Department of Plastic and Reconstructive Surgery, Kangnam Sacred Heart Hospital, Hallym University College of Medicine, Seoul 07247, Republic of Korea; ssw1218@gmail.com (S.S.W.);; 3 Department of Surgery, Incheon St. Mary’s Hospital, College of Medicine, The Catholic University of Korea, Seoul 06591, Republic of Korea; 4 Division of Breast Surgery, Department of Surgery, Yonsei University College of Medicine, Seoul 03186, Republic of Korea; 5 Department of Surgery, Seoul National University Bundang Hospital, Seoul National University College of Medicine, Seongnam 13620, Republic of Korea; 6 Department of Surgery, Jeonbuk National University Medical School, Jeonju-si 54907, Republic of Korea; yhj0903@jbnu.ac.kr; 7 Division of Breast and Endocrine Surgery, Department of Surgery, Korea University Anam Hospital, Korea University College of Medicine, Seoul 03080, Republic of Korea

**Keywords:** fine needle aspiration, core needle biopsy, palpable tumor, breast cancer, BIRADS

## Abstract

**Simple Summary:**

Core needle biopsy (CNB) and fine needle aspiration (FNA) are the most commonly used non-surgical tissue sampling methods for breast cancer diagnosis. CNB has higher diagnostic accuracy and enables molecular subtype determination for neoadjuvant chemotherapy, and is more widely used than FNA. However, FNA is less invasive and provides faster results, and is still performed by many clinicians. This study was conducted to investigate the prognosis and application criteria of the two methods in real clinical practice. We found that patients who were diagnosed with breast cancer using FNA had significantly worse survival rates than those diagnosed using CNB. In the subgroup analysis, FNA showed worse survival rates in cases of highly suspicious lesions, nonpalpable tumors, or centrally located tumors. Our study may help in choosing the appropriate tissue sampling method for suspected breast cancer cases.

**Abstract:**

(1) Background: Breast core needle biopsy (CNB) is preferred over fine needle aspiration (FNA) as it has higher sensitivity and specificity and enables immunohistochemical evaluation. However, breast FNA remains widely used because of its low cost, minimally invasive nature, and quick results. Studies analyzing the effects of each test on the prognoses of patients with breast cancer are scarce and controversial, and the criteria for test selection remain unknown. (2) Methods: This study included adult female patients who underwent breast cancer surgery at 102 general hospitals. The trend of breast biopsies over time was analyzed, and the prognoses of patients with breast cancer who underwent CNB and FNA were compared. (3) Results: This study included 73,644 patients who underwent FNA (*n* = 8027) and CNB (*n* = 65,617). A multivariate Cox regression analysis showed that patients diagnosed using FNA had significantly worse overall survival (OS) and breast-cancer-specific survival (BCSS) than those diagnosed using CNB. In the subgroup analysis, patients with breast imaging reporting and data system (BI-RADS) 5 lesions, palpable tumors, or centrally located tumors had significantly worse OS and BCSS with FNA than with CNB. (4) Conclusions: CNB should be performed preferentially instead of FNA in patients with BI-RADS 5 lesions and nonpalpable or centrally located tumors.

## 1. Introduction

Breast cancer is the most common cancer among women, accounting for almost one-third of all cancers in women [1]. It is the leading cause of cancer-related mortality in women aged 20–59 years, and its incidence has been increasing [1]. Patients with symptoms or risk factors of breast cancer experience high anxiety [2]. Breast diseases can be diagnosed quickly and accurately using a triple diagnostic assessment, including clinical, imaging, and pathological tests [3].

Fine needle aspiration (FNA) and core needle biopsy (CNB) are the most commonly used nonoperative pathological tests for breast lesions and both have high sensitivity and specificity. FNA uses a smaller, solid needle (usually 25-gauge) that can only sample some cells from the breast mass; CNB uses a larger, hollow needle (usually 14-gauge) that can sample a small cylinder of tissue (core) from the breast mass. Breast FNA has a lower diagnostic performance than CNB and cannot identify prognostic/predictive biomarkers. The accuracy of preoperative breast biopsy varies greatly across different studies, especially for FNA. In several studies, the sensitivity of FNA (35–95%) showed greater variability and was generally lower than that of CNB (85–100%). Furthermore, the specificity of FNA (48–100%) was also generally lower than that of CNB (86–100%) [4].

Hence, many clinicians prefer CNB as the biopsy method. The ratio of CNB is increasing every year and the ratio of FNA is decreasing in East Asia [5,6]. However, FNA has high sensitivity and specificity and is less invasive when performed by an experienced physician. Furthermore, it reduces patient anxiety due to its less invasive nature and provides quick results at a lower cost [7]. Hence, FNA is still performed as a routine test in many institutions in the United States of America and Europe [8].

Many studies have compared the accuracy of breast CNB and FNA [4,9,10,11,12]. However, studies investigating the effects of CNB and FNA on the prognoses of patients with breast cancer are few, and their results are controversial. CNB is more invasive than FNA and is more likely to induce tissue inflammation or tract seeding. Inflammation caused by mechanical stress generated through breast biopsy affects breast cancer development and axillary lymph node dissemination [13,14,15,16,17]. However, some studies have reported that biopsies do not affect local recurrence or prognosis [9,18]. Breast FNA tends to be used for lesions with a clinically low suspicion of breast cancer. However, there are no clearly defined criteria for biopsy in clinical practice [7].

Thus, this study aimed to investigate trends in biopsy use and the effect of biopsy methods on the prognoses of patients with breast cancer and determine whether an appropriate test could be selected according to patients’ clinical and radiological features.

## 2. Materials and Methods

### 2.1. Patients

This retrospective study was based on prospectively collected nationwide multicenter data from patients diagnosed with breast cancer at 102 general hospitals in South Korea. The Korean Breast Cancer Society collected all patient data, and all patients provided written informed consent for storing and using their information for research purposes. The study participants were adult women diagnosed with invasive breast cancer who underwent surgery between 2004 and 2013. Patients whose diagnostic tools were unclear or were diagnosed using methods other than FNA or CNB were excluded. The patients included in the final analysis were divided into two groups: those diagnosed using CNB (the CNB group) and those diagnosed using FNA (the FNA group). The study protocol was approved by the Institutional Review Board of the Catholic University of Korea (VC22ZISI0018).

### 2.2. Definitions

Human epidermal growth factor receptor 2 (HER2) positivity was defined as either 3+ overexpression or HER2 amplification observed via immunohistochemical staining or fluorescent in situ hybridization, respectively (HER2/chromosome enumeration probe 17 ratios: ≥2.0). Hormone receptor (HR) positivity was defined as >1% staining for either estrogen or progesterone receptors or both. Breast radiologists categorized the tumors according to the breast imaging reporting and data system (BI-RADS) categories and recorded them according to the fourth edition of the Atlas. The BI-RADS was published by the American College of Radiology to provide standardized breast imaging terminology in report organization, assessment structure, and classification systems for mammography, ultrasound, and magnetic resonance imaging of the breast [19].

### 2.3. Statistical Analysis

The characteristics of patients in the FNA and CNB groups were compared using the Chi-square test and Student’s *t*-test for continuous and non-continuous variables, respectively. Survival graphs were drawn using Kaplan–Meier estimates, and hazard ratios were calculated using Cox regression analysis. Multivariate Cox proportional hazards regression analysis included statistically significant variables from the univariate analysis as covariates. The analyses were performed using R software (ver. 4.2.2, R Core Team, 2022).

### 2.4. Subgroup Analysis

Univariate and multivariate Cox regression analyses were used to determine whether the diagnostic methods affected prognosis according to the patient’s age, tumor palpability, location, size, and BI-RADS score, which can be confirmed before the biopsy.

## 3. Results

### 3.1. Time Trends in Breast Biopsy Practice

**Figure 1:**
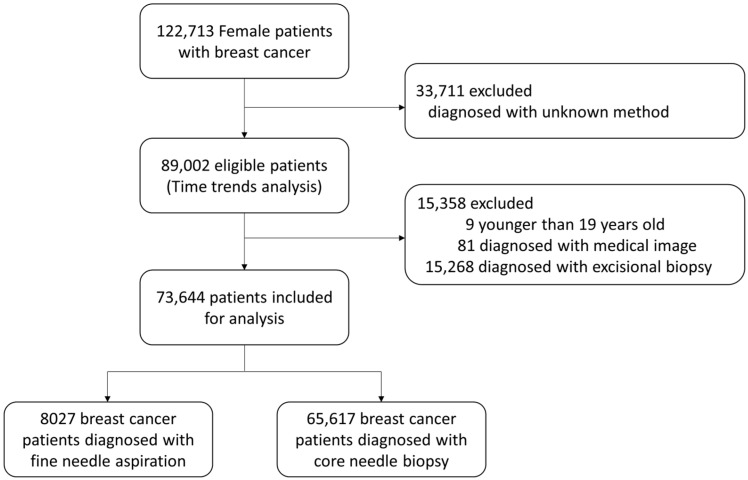
Flow diagram of study patient selection from the Korean Breast Cancer Society database.

**Figure 2:**
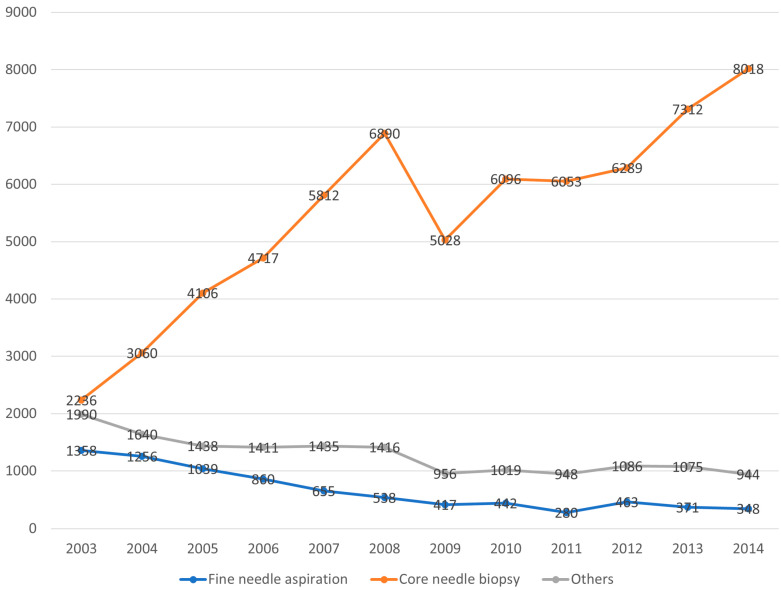
Time trends in breast biopsy practice.

From 2003 to 2014, 89,002 women were diagnosed with breast cancer, and the time trends of breast biopsies were analyzed for this patient group (Figure 1). In 2003, 24.3% of all diagnoses were made using FNA. However, the use of FNA decreased over time, accounting for 3.7% of diagnoses in 2014. In contrast, CNB use increased from 40.0% in 2003 to 86.1% in 2014, accounting for most breast cancer diagnoses (Figure 2).

### 3.2. Patient Characteristics

Among the 89,002 patients, 73,644 who underwent FNA and CNB were included in the final analysis, and 65,317 (89.1%) were diagnosed with breast cancer using CNB (Figure 1). The median follow-up time of the patients was 84.1 ± 31.4 months after diagnosis. The age difference between the two groups was not significant (FNA: 50.1 ± 10.7 years vs. CNB: 50.3 ± 10.5 years). Patients in the FNA group had significantly larger tumors (2.5 ± 3.1 cm vs. 2.0 ± 2.9 cm), more palpable tumors (92.6% vs. 81.7%), more frequent lymph node metastases (41.6% vs. 33.8%), higher histologic grades, and more cases of mastectomy (53.5% vs. 40.7%). The patients’ characteristics are summarized in Table 1.

### 3.3. Survival Analysis

**Figure 3:**
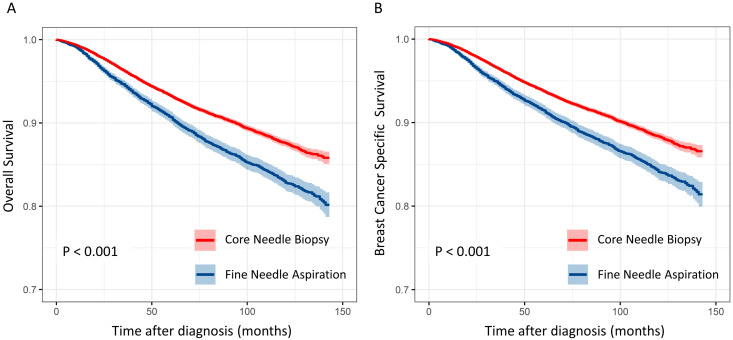
Comparative graph of the survival rates of breast cancer patients diagnosed using core needle biopsy and fine needle aspiration. (**A**) Overall survival; (**B**) Breast cancer specific survival.

The CNB group had significantly more favorable overall survival (OS) and breast-cancer-specific survival (BCSS) (Figure 3). Univariate analysis showed that age, surgery type, stage, location, palpation, histologic grade, HR status, HER2 status, and biopsy method significantly affected survival rate. Both univariate and multivariate analyses showed that the CNB group had significantly better OS and BCSS rates than the FNA group (univariate Cox regression: OS 1.415 [1.322–1.515], BCSS 1.342 [1.249–1.443] vs. multivariate Cox regression: OS 1.123 [1.026–1.228], BCSS 1.099 [1.001–1.206]; Table 2 and Table 3).

**Figure 4:**
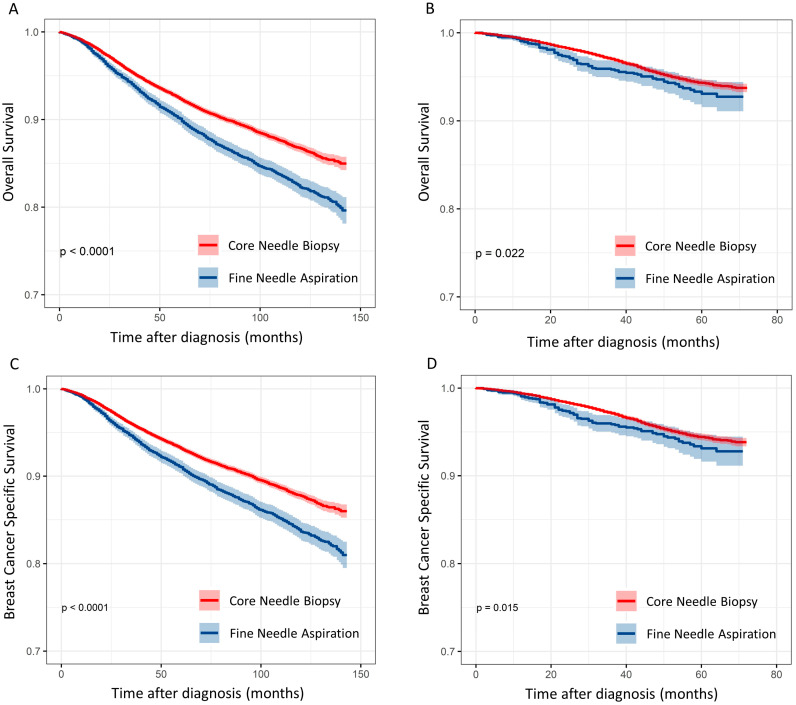
Survival graphs using Kaplan–Meier estimates (**A**,**C**) 2004–2009 and (**B**,**D**) 2010–2014.

When data for 2004–2009 and 2010–2014 were analyzed separately, the FNA group had significantly worse survival rates than the CNB group in both periods (Figure 4).

### 3.4. Subgroup Analysis

In the subgroup analysis for age and tumor size, the FNA group showed significantly worse OS and BCSS than the CNB group in the univariate analysis. However, no significant difference was observed in the multivariate analysis. Additionally, the analysis for BI-RADS 5 lesions and nonpalpable and centrally located breast cancers showed that the FNA group had significantly poorer OS and BCSS than the CNB group in the univariate and multivariate analyses.

The subgroup analysis is described in Table 4 and Table 5.

## 4. Discussion

FNA and CNB are widely used techniques and have their strengths and weaknesses. Although the selection criteria for biopsies have not been established, the frequency of selecting CNB with better performance is much higher, and this trend continues to increase [11]. FNA and CNB are sometimes used simultaneously for higher sensitivity, specificity, and negative predictive value; however, this does not commonly occur [8,12]. This study reported that 73.4% of patients with breast cancer were diagnosed using CNB; additionally, this proportion was confirmed to be increasing.

FNA differs in adequacy, sensitivity, and specificity according to the skill level of clinicians and pathologists. FNA biopsy can maintain accuracy when clinicians and pathologists implement quality control through continuous training and rapid on-site evaluation [20,21,22,23]. However, learning opportunities for performing and interpreting FNA are simultaneously decreasing with the frequency of use of breast FNA [20,24].

There are differences in FNA methods, pathological analysis, and reporting systems among institutions. For example, only 54.5% of the laboratories enrolled in the 2019 College of American Pathologists Non-Gynecologic Cytopathology Education Program used a standardized reporting system. In addition, significant differences were found in primary slide preparation methods, ancillary studies, fixation time reporting, standardized reporting systems, and the descriptive diagnosis of breast FNA [8]. Furthermore, testing for prognostic/predictive biomarkers using FNA is impossible; hence, it cannot be used in neoadjuvant settings. It is also difficult to differentiate ductal carcinoma in situ due to the cytological features of FNA that do not require invasion [4]. Diagnostic delays due to the disadvantages of FNA mentioned above may have adversely affected the prognoses of patients who underwent FNA in the present study.

Previous studies that analyzed the effects of CNB and FNA on the prognosis of patients with breast cancer are controversial. Kong et al. reported that the prognosis of patients with breast cancer who did not receive radiotherapy (RT) after CNB was poorer than that of patients who received RT due to cancer cell tract seeding that can occur during CNB [10]. Although patients who did not undergo RT were included in this study, CNB showed a better prognosis than FNA.

To the best of our knowledge, this is the first study to show that the selection of a biopsy method based on patients’ clinicopathological features influences the prognosis of patients with breast cancer. In clinical practice, FNA is usually performed on lesions with a low suspicion of breast cancer [7]. In this study, for BI-RADS 0–4 lesions, there was no difference in the prognosis between patients who underwent CNB and those who underwent FNA. In comparison, for BI-RADS 5 lesions, patients who underwent FNA showed a significantly poorer prognosis. Thus, CNB, rather than FNA, may result in a better prognosis for highly suspicious breast nodules in patients with breast cancer.

FNA has a higher inadequacy rate with nonpalpable masses and shows poorer performance than CNB [25,26]. FNA is performed using a thin needle, and the number of cells that can be obtained is smaller than that with CNB; hence, accurate localization is important. The firm breast tissue is thick in the central portion of the breast, making precise localization more difficult with a fine needle. This study confirmed that the prognosis after FNA was poorer than that after CNB in patients with nonpalpable and centrally located masses, which may be related to the test’s accuracy.

This study had some limitations. First, this was a retrospective study. Therefore, selection bias may exist, and the causal relationship between the test accuracy and survival rate could not be determined. Second, the FNA proportion among the biopsy methods was higher in the past. A decrease in breast cancer mortality due to advances in treatment may have contributed to improved prognoses over time and poorer prognoses after FNA in the past [27]. However, in this study, FNA showed a poorer prognosis than CNB before and after 2009. Third, this study did not include a subgroup analysis of invasive micropapillary carcinoma or DCIS, which are known to have different imaging aspects and be more difficult to distinguish using fine needle aspiration [28,29,30].

## 5. Conclusions

Patients with breast cancer who underwent FNA as a primary biopsy had poorer prognoses than those who underwent CNB for highly suspicious, nonpalpable, and centrally located breast nodules. Hence, performing CNB before FNA is a promising approach for achieving a good prognosis. Further prospective studies are warranted to investigate the direct relationship between biopsy method accuracy and prognosis.

## Figures and Tables

**Table 1:** Clinical and histopathological characteristics (*n* = 73,644).

Characteristics	Biopsy Method (*n* = 73,644)		
	FNA (*n* = 8027)	CNB (*n* = 65,617)	*p*-Value
Age, years (mean ± SD)	50.1 ± 10.7	50.3 ± 10.5	0.127
Age, years *n* (%)			0.368
<55	5633 (70.2)	45,923 (70.0)	
≥55	2394 (29.8)	19,694 (30.0)	
Tumor size (cm)	2.5 ± 3.1	2.0± 2.9	<0.001
Nodal involvement *n* (%)			<0.001
No	4633 (58.4)	43,011 (66.2)	
Yes	3305 (41.6)	21,948 (33.8)	
Unknown	89	658	
Stage *n* (%)			<0.001
I	2377 (31.7)	25,657 (43.6)	
II	3512 (46.9)	23,828 (40.4)	
III	1484 (19.8)	8562 (14.5)	
IV	115 (1.5)	863 (1.5)	
Palpation *n* (%)			<0.001
Yes	6839 (92.6)	45,458 (81.7)	
No	543 (7.4)	10,162 (18.3)	
Unknown	645	9997	
Tumor Count *n* (%)			<0.001
Single	6074 (84.7)	54,384 (87.2)	
Multiple	1100 (15.3)	7515 (12.8)	
Type *n* (%)			<0.001
IDC	6984 (93.3)	54,906 (93.2)	
ILC	144 (1.9)	1995 (3.4)	
Others	360 (438)	2009 (3.4)	
Location *n* (%)			<0.001
Lateral	4530 (62.5)	38,193 (64.8)	
Medial	1742 (24.0)	14,797 (25.1)	
Central	977 (13.5)	5953 (10.1)	
Unknown	2346	6674	
Histologic grade *n* (%)			<0.001
Grades 1 and 2	3586 (54.7)	34,976 (63.9)	
Grade 3	2970 (45.3)	19,755 (36.1)	
Unknown	1471	10,886	
Surgery			<0.001
Breast conserving	3531 (44.0%)	37,791 (57.6)	
Mastectomy	4292 (53.5%)	26,697 (40.7)	
Other	204 (2.5)	1129 (1.7)	
BI-RADS category *n* (%)			<0.001
0–3	187 (3.0)	1036 (2.0)	
4	369 (6.0)	6451 (12.5)	
5	5600 (91.0)	44,282 (85.5)	
Hormonal receptor *n* (%)			<0.001
Positive	5331 (68.7)	47,523 (74.2)	
Negative	2429 (31.3)	16,506 (25.8)	
Unknown	267	1588	
HER2 *n* (%)			<0.001
Positive	1667 (28.6)	13,144 (25.9)	
Negative	4158 (71.4)	37,647 (74.1)	
Unknown	4902	14,826	

Groups were compared using analysis of variance (*t*-test), and continuous variables were analyzed using the Chi-square test. BI-RADS = breast imaging reporting and data system; CNB = core needle biopsy; FNA = fine needle aspiration; IDC = invasive ductal carcinoma; ILC = invasive lobular carcinoma; HER2 = human epidermal growth factor receptor 2.

**Table 2:** Univariate and multivariate analyses of prognostic factors for overall survival.

	Univariate Analysis	*p*-Value	Multivariate Analysis	*p*-Value
Age				
<55	1		1	
≥55	1.637 (1.547–1.733)	<0.001	1.545 (1.429–1.670)	<0.001
Surgery				
Breast conserving	1		1	
Mastectomy	2.722 (2.558–2.897)	<0.001	1.517 (1.386–1.661)	<0.001
Others	4.614 (4.022–5.293)	<0.001	2.222 (1.699–2.907)	<0.001
Tumor type				
IDC	1		1	
ILC	0.924 (0.773–1.104)	0.219	1.172 (0.884–1.554)	0.269
Others	1.677 (1.479–1.901)	0.060	1.316 (1.813–0.983)	0.039
Stage				
I	1		1	
II	2.264 (2.081–2.464)	<0.001	1.751 (1.559–1.967)	<0.001
III	7.334 (6.747–7.971)	<0.001	5.192 (4.610–5.846)	<0.001
IV	28.587 (25.488–32.063)	<0.001	18.795 (15.629–22.603)	<0.001
Location				
Peripheral	1		1	
Central	1.938 (1.804–2.082)	<0.001	1.271 (1.153–1.400)	<0.001
Palpation				
No	1		1	
Yes	2.283 (2.008–2.595)	<0.001	1.215 (1.012–1.459)	0.001
Tumor count				
Single	1		1	
Multiple	1.272 (1.176–1.375)	0.260	1.018 (0.914–1.134)	0.743
Histologic grade				
G 1–2	1		1	
G 3	2.084 (1.964–2.212)	<0.001	1.531 (1.411–1.661)	<0.001
BI-RADS				
0–3	1		1	
4	0.648 (0.504–0.834)	0.001	1.040 (0.742–1.459)	0.819
5	1.125 (0.921–1.375)	0.250	1.146 (0.866–1.516)	0.342
Biopsy				
CNB	1		1	
FNA	1.415 (1.322–1.515)	<0.001	1.123 (1.026–1.228)	0.012
Estrogen Receptor				
Positive	1		1	
Negative	1.934 (1.828–2.046)	<0.001	1.603 (1.483–1.733)	<0.001
HER2				
Negative	1		1	
Positive	1.261 (1.178–1.349)	<0.001	1.100 (1.016–1.191)	0.019

BI-RADS = breast imaging reporting and data system; CNB = core needle biopsy; FNA = fine needle aspiration; IDC = invasive ductal carcinoma; ILC = invasive lobular carcinoma; HER2 = human epidermal growth factor receptor 2.

**Table 3:** Univariate and multivariate analyses of prognostic factors for breast-cancer-specific survival.

	Univariate Analysis	*p*-Value	Multivariate Analysis	*p*-Value
Age				
<55	1		1	
≥55	1.506 (1.419–1.59)	<0.001	1.439 (1.332–1.555)	<0.001
Surgery				
Breast conserving	1		1	
Mastectomy	2.771 (2.597–2.957)	<0.001	1.509 (1.378–1.652)	<0.001
Others	4.755 (4.127–5.478)	<0.001	2.216 (1.772–2.772)	<0.001
Tumor type				
IDC	1		1	
ILC	0.889 (0.738–1.072)	0.219	0.952 (0.744–1.220)	0.700
Others	1.236 (0.991–1.542)	0.060	1.323 (0.938–1.866)	0.111
Stage				
I	1		1	
II	2.452 (2.238–2.686)	<0.001	2.020 (1.793–2.277)	<0.001
III	8.416 (7.697–9.203)	<0.001	6.474 (5.741–7.300)	<0.001
IV	33.503 (29.728–37.757)	<0.001	24.706 (20.789–29.359)	<0.001
Location				
Peripheral	1		1	
Central	2.006 (1.863–2.159)	<0.001	1.267 (1.153–1.393)	<0.001
Palpation				
Yes	1		1	
No	2.323 (2.031–2.657)	<0.001	1.264 (1.053–1.519)	0.012
Tumor count				
Single	1		1	
Multiple	1.311 (1.210–1.421)	<0.001	1.066 (0.960–1.183)	0.231
Histologic grade				
G 1–2	1		1	
G 3	2.187 (2.055–2.326)	<0.001	1.576 (1.448–1.715)	<0.001
BI-RADS				
0–3	1		1	
4	0.666 (0.513–0.863)	0.002	1.068 (0.754–1.514)	0.711
5	1.123 (0.912–1.383)	0.274	1.135 (0.850–1.515)	0.390
Biopsy				
CNB	1		1	
FNA	1.342 (1.249–1.443)	<0.001	1.099 (1.001–1.206)	0.047
Estrogen Receptor				
Positive	1		1	
Negative	2.003 (1.888–2.124)	<0.001	1.668 (1.531–1.816)	<0.001
HER2				
Negative	1		1	
Positive	1.354 (1.261–1.454)	<0.001	1.081 (0.992–1.180)	0.077

BI-RADS = breast imaging reporting and data system; CNB = core needle biopsy; FNA = fine needle aspiration; IDC = invasive ductal carcinoma; ILC = invasive lobular carcinoma; HER2 = human epidermal growth factor receptor 2.

**Table 4:** Subgroup analysis of hazard ratios of overall survival using Cox regression.

	CNB	FNA (Univariate)	*p*-Value	FNA (Multivariate)	*p*-Value
Age (years)					
<55	1	1.396 (1.278–1.524)	<0.001	1.083 (0.968–1.212)	0.165
≥55	1	1.410 (1.265–1.572)	<0.001	1.146 (0.994–1.321)	0.061
Size					
<2 cm	1	1.385 (1.213–1.582)	<0.001	1.134 (0.947–1.358)	0.171
2–4 cm	1	1.216 (1.097–1.349)	<0.001	1.073 (0.943–1.220)	0.283
≥4 cm	1	1.257 (1.107–1.428)	<0.001	1.135 (0.963–1.338)	0.132
Location					
Peripheral	1	1.386 (1.280–1.501)	<0.001	1.066 (0.964–1.179)	0.213
Central	1	1.395 (1.200–1.621)	<0.001	1.309 (1.089–1.574)	0.004
BI-RADS					
0–3	1	1.726 (1.116–2.672)	0.014	1.180(0.667–2.090)	0.569
4	1	1.629 (1.031–2.575)	0.037	0.881 (0.450–1.723)	0.711
5	1	1.338 (1.239–1.445)	<0.001	1.122 (1.025–1.228)	0.012
Palpable					
Yes	1	1.488 (0.986–2.247)	0.059	0.963 (0.557–1.666)	0.893
No	1	1.302 (1.211–1.400)	<0.001	1.224 (1.126–1.331)	<0.001

CNB = core needle biopsy; FNA = fine needle aspiration; BI-RADS = breast imaging reporting and data system.

**Table 5:** Subgroup analysis of hazard ratios of breast-cancer-specific survival using Cox regression.

	CNB	FNA (Univariate)	*p*-Value	FNA (Multivariate)	*p*-Value
Age (years)					
<55	1	1.336 (1.219–1.463)	<0.001	1.045 (0.949–1.151)	0.374
≥55	1	1.330 (1.181–1.498)	<0.001	1.113 (0.981–1.264)	0.097
Size					
<2 cm	1	1.287 (1.113–1.488)	0.001	1.104 (0.950–1.282)	0.199
2–4 cm	1	1.149 (1.030–1.281)	0.013	1.020 (0.912–1.140)	0.731
≥4 cm	1	1.205 (1.057–1.374)	0.005	1.105 (0.966–1.262)	0.145
Location					
Peripheral	1	1.312 (1.206–1.428)	<0.001	1.049 (0.961–1.146)	0.283
Central	1	1.323 (1.130–1.547)	<0.001	1.201 (1.018–1.416)	0.030
BI-RADS					
0–3	1	1.708 (1.082–2.698)	0.022	1.221 (0.677–2.199)	0.507
4	1	1.366 (0.822–2.269)	0.228	1.191 (0.711–1.995)	0.507
5	1	1.274 (1.175–1.383)	<0.001	1.113 (1.023–1.211)	0.013
Palpable					
Yes	1	1.218 (0.751–1.975)	0.424	0.932 (0.557–1.559)	0.788
No	1	1.266 (1.173–1.367)	<0.001	1.120 (1.034–1.212)	0.005

CNB = core needle biopsy; FNA = fine needle aspiration; BI-RADS = breast imaging reporting and data system.

## Data Availability

The data presented in this study are available on request from the corresponding author.
